# The knowledge driven DBTL cycle provides mechanistic insights while optimising dopamine production in *Escherichia coli*

**DOI:** 10.1186/s12934-025-02729-6

**Published:** 2025-05-16

**Authors:** Lorena Hägele, Natalia Trachtmann, Ralf Takors

**Affiliations:** 1 Institute of Biochemical Engineering, University of Stuttgart, Allmandring 31, 70569 Stuttgart, Germany; 2 Laboratory of Molecular Genetics and Microbiology Methods, Kazan Scientific Center of Russian Academy of Sciences, 420111 Kazan, Russia

**Keywords:** Dopamine, DBTL cycle, Biofoundry, Crude cell lysates, *Escherichia coli*

## Abstract

**Background:**

Dopamine is a promising organic compound with several key applications in emergency medicine, diagnosis and treatment of cancer, production of lithium anodes, and wastewater treatment. Since studies on in vivo dopamine production are limited, this study demonstrates the development and optimisation of a dopamine production strain by the help of the knowledge driven design-build-test-learn (DBTL) cycle for rational strain engineering.

**Results:**

The knowledge driven DBTL cycle, involving upstream in vitro investigation, is an automated workflow that enables both mechanistic understanding and efficient DBTL cycling. Following the in vitro cell lysate studies, the results were translated to the in vivo environment through high-throughput ribosome binding site (RBS) engineering. As a result, we developed a dopamine production strain capable of producing dopamine at concentrations of 69.03 ± 1.2 mg/L which equals 34.34 ± 0.59 mg/g_biomass_. Compared to state-of-the-art in vivo dopamine production, our approach improved performance by 2.6 and 6.6-fold, respectively.

**Conclusion:**

In essence, a highly efficient dopamine production strain was developed by implementing the knowledge driven DBTL cycle involving upstream in vitro investigation. The fine-tuning of the dopamine pathway by high-throughput RBS engineering clearly demonstrated the impact of GC content in the Shine-Dalgarno sequence on the RBS strength.

**Supplementary Information:**

The online version contains supplementary material available at 10.1186/s12934-025-02729-6.

## Background

Dopamine (3,4-dihydroxyphenethylamine) is a promising organic compound in the catecholamine and phenethylamine families, has key applications in emergency medicine for regulating blood pressure, renal function, and neurobehavioral disorders [1, 2]. Under alkaline conditions, it can self-polymerize into biocompatible polydopamine [3], which is applicable in the diagnosis and treatment of cancer [4], in agriculture for plant protection [5], in wastewater treatment to remove heavy metal ions and organic contaminants [3, 6–8], and in the production of lithium anodes in fuel cells as a strong ion and electron conductor [9–11]. Large-scale production of dopamine is currently achieved through chemical synthesis [12, 13] or enzymatic systems [14], both of which are environmentally harmful and resource-intensive. Dong et al. have developed an environmentally friendly method for synthesising dopamine hydrochloride [2].

**Fig. 1:**
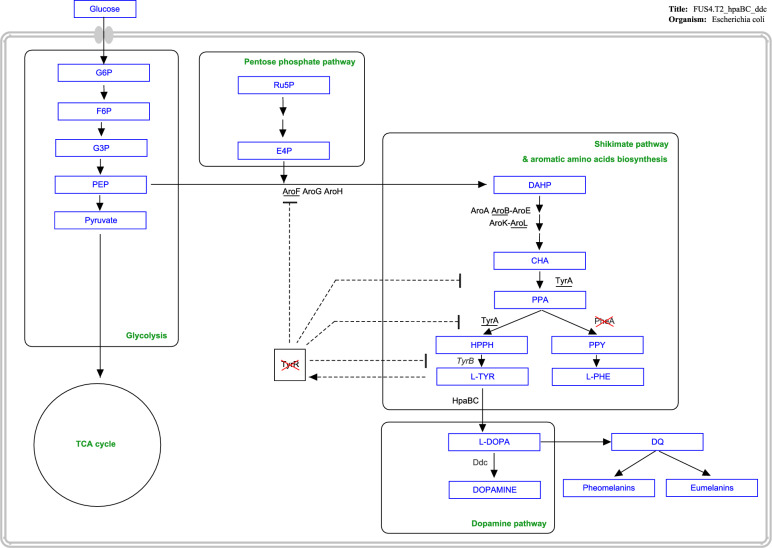
Pathways involved in dopamine biosynthesis and regulation in *E. coli*. Also, the strain engineering targets are shown either by a red cross to indicate deletion of *tyrR* or by underline to indicate overexpression of a gene (*tyrA*, *aroF*, *aroB*, *aroL*). Dashed lines indicate feedback inhibition. G6P: Glucose-6-phosphate; F6P: Fructose-6-phosphate; G3P: Glycerinaldehyde-3-phosphate; PEP: Phosphoenolpyruvate; TCA: Tricarboxylic acid; Ru5P: Ribulose-5-phosphate; E4P: Erythrose-4-phosphate; DAHP: 3-Desoxyarabinoheptulosanat-7-phosphate; CHA: Chorismate; PPA: Prephenate; HPPH: 4-Hydroxyphenylpyruvate; L-TYR: l-tyrosine; PPY: phenylpyruvate; L-PHE: l-phenylalanine; AroF/AroG/AroH: 3-Deoxy-D-arabinoheptulosonat-7-phosphate-synthase; AroA: 5-Enolpyruvoyl-shikimate-3-phosphate-synthase; AroB: 3-Dehydroquinat-synthase; AroK/ AroL: Shikimate-kinase 1 + 2; l-DOPA: 3;4-Dihydroxy-phenylalanin; DQ: Dopaquinone; TyrR: transcriptional regulator; PheA/ TyrA: Chorismate-mutase; TyrB: Tyrosine aminotransferase; HpaBC: 4-hydroxyphenylacetate 3-monooxygenase; Ddc: l-DOPA decarboxylase. The schematic graph was created using PathVisio3 software [61]

In addition to those dopamine production methods, in vivo production of dopamine in *Escherichia coli* (*E. coli*) have been established, starting with l-tyrosine as the precursor (Fig. 1). The native *E. coli* gene encoding 4-hydroxyphenylacetate 3-monooxygenase (HpaBC) converts l-tyrosine to l-DOPA [15]. Subsequently, l-DOPA decarboxylase (Ddc) from *Pseudomonas putida* catalyses the formation of dopamine [16]. Whereas l-DOPA synthesis is well studied [17–20], studies on in vivo dopamine production are limited [12, 21], with reported maximum production titres of 27 mg/L and 5.17 mg/g_biomass_. Genomic engineering of *E. coli* is essential to achieve increased l-tyrosine concentrations, as l-tyrosine is the precursor of l-DOPA and dopamine (Fig. 1). The depletion of the transcriptional dual regulator l-tyrosine repressor TyrR [22] and the mutation of the feedback inhibition of chorismate mutase/ prephenate dehydrogenase (*tyrA*) [23] could increase l-tyrosine production [18, 20]. Developing a dopamine production strain is thus a promising approach when targeting dopamine synthesis.

To enhance the efficiency of strain construction, as well as the evaluation of dopamine production host, the application of the design-build-test-learn (DBTL) cycle is well suited [24]. Modular design tools, data management systems, and models have been integrated into the DBTL cycle to support the initial design phase [25, 26]. The build and testing phases, which involve DNA assembly, molecular cloning, and strain analysis, are becoming increasingly automated with advanced genetic engineering tools [25, 27–31]. Finally, the learning phase incorporates both traditional statistical evaluations and model-guided assessments, including machine learning techniques, to refine strain performance [32, 33]. The full automation of DBTL cycles, known as biofoundries [34, 35], are becoming central to synthetic biology. One major challenge in DBTL cycles is the entry point, as the initial round typically starts without prior knowledge. Besides biofoundry approaches, rational design [36] and hypothesis driven design [37, 38] are the main strategies used to select engineering targets. However, in most DBTL cycles, engineering targets are selected via design of experiment [24, 39–41] or randomised selection [42], which can lead to more iterations and extensive consumption of time, money and resources. To address this, we adopted a mechanistic rather than statistical approach, conducting in vitro tests to assess enzyme expression levels in the dopamine production host before DBTL cycling, similar to Dudley et al. and Karim et al. [43, 44]. We will refer to this process as the knowledge driven DBTL cycle.

In vitro protein synthesis is a promising format for producing biotechnological products such as l-malate [45] and synephrine from l-tyrosine [46]. Cell-free protein synthesis (CFPS) systems are particularly useful for bypassing whole-cell constraints such as membranes and internal regulation [47]. Crude cell lysate systems are especially advantageous, as they ensure supply of e.g. metabolites and energy equivalents [48, 49]. Both approaches were applied by Karim et al. to design and optimise metabolic pathways in *Clostridium autoethanogenum* [44]. However, the described work only focused on metabolic pathway expression in *Clostridium autoethanogenum* and did not consider further in vivo fine-tuning. Our approach leverages in vitro crude cell lysate systems to test different relative expression levels, accelerating strain development in *E. coli*. To achieve efficient strain construction through the DBTL cycle, a range of tools are available to translate the different relative expression levels into the in vivo environment, including ribosome binding site (RBS) engineering and promoter engineering [39]. We chose RBS engineering for precise fine-tuning of our dopamine production strains.

RBS engineering is a powerful technique to fine-tune relative gene expression in synthetic pathways [50–53], with tools like the untranslated region (UTR) Designer for modulating RBS sequences [54]. However, these tools often focus on flanking regions of the Shine-Dalgarno (SD) sequence, although the complex region is crucial for secondary structures of the RBS [53–56]. Simplified RBS engineering can be achieved by modulating the SD sequence without interfering the secondary structure [57, 58]. Whereas RBS characterisation typically involves single genes to assess translation initiation rate (TIR) or Gibbs free energy [54, 57, 59], only a few studies have explored polycistronic pathways for compound production, such as violacein [60]. These studies have generally been non- or semi-automated, with automation mainly limited to the cultivation process [52, 59, 60]. The knowledge driven DBTL cycle aims to utilise bi-cistronic gene expression to build an efficient synthetic pathway for dopamine production.

This study demonstrates the development and optimisation of a dopamine production strain with the help of the knowledge driven DBTL cycle for rational strain engineering. By combining in vitro pathway design with high-throughput in vivo RBS engineering, dopamine production was increased. A host strain was engineered for high l-tyrosine production, enabling efficient dopamine synthesis.

## Materials and methods

### Media and buffer solutions

2xTY medium was prepared as described previously [38]. SOC medium was prepared by adding 5 g/L yeast extract, 20 g/L tryptone, 10 mM NaCl, 2.5 mM KCl, 10 mM MgCl_2_ and 10 mM MgSO_4_ dissolved in deionised water. After autoclaving, glucose was added from a sterile stock to a final concentration of 20 mM.

The minimal medium for cultivation experiments consisted of 20 g/L glucose, 10% 2xTY medium, 2.0 g/L NaH_2_PO_4_⋅2H_2_O, 5.2 g/L K_2_HPO_4_, 4.56 g/L (NH_4_)_2_SO_4_, 15 g/L 3-(N-morpholino) propanesulfonic acid (MOPS), 50 µM vitamin B6, 5 mM phenylalanine, 0.2 mM FeCl_2_, and 0.4% (V/V) trace element stock solution. The composition of the trace element stock solution was 4.175 g/L FeCl_3_⋅6H_2_O, 0.045 g/L ZnSO_4_⋅7H_2_O, 0.025 g/L MnSO_4_⋅H_2_O, 0.4 g/L CuSO_4_⋅5H_2_O, 0.045 g/L CoCl_2_⋅6H_2_O, 2.2 g/L CaCl_2_⋅2H_2_O, 50 g/L MgSO_4_⋅7H_2_O, and 55 g/L sodium citrate dehydrate. Stock solutions of salts, trace elements and sugars were autoclaved separately, and stock solutions of antibiotics were filter sterilised and stored at − 20 °C. All compounds were combined shortly before the experiments to avoid possible ageing of the medium. Appropriate antibiotics and inducers were added to the liquid medium and agar plates at the following concentrations: ampicillin 100 µg/mL, kanamycin 50 µg/mL, and isopropyl β-d-1-thiogalactopyranoside (IPTG) 1 mM.

Phosphate buffer 50 mM at pH 7 was prepared by adding 28.9 mL of 1 M KH_2_PO_4_ and 21.1 mL of 1 M K_2_HPO_4_ stock solution to 1 L of deionised water, adjusting the pH by adding KOH and then autoclaving the buffer. Reaction buffer for the crude cell lysate system was prepared by adding 0.2 mM FeCl_2_, 50 µM vitamin B6, and 1 mM l-tyrosine or 5 mM l-DOPA to phosphate buffer. To prepare the concentrated reaction buffer, a fivefold amount of the supplements was used.

### Bacterial strains, genes and plasmids

The bacterial strains and plasmids used in this study are listed in Tables 1 and 2. The Primers are listed in supplementary information S1. The DNA sequence of the heterologous expressed genes *hpaBC* and *ddc* is given in supplementary information S2. *E. coli* DH5α was used as the cloning strain and *E.* *coli* FUS4.T2 was used as the production strain.

**Table 1:** Strains used in this study

Strain	Genotype/ Strain information	References/Source
*E. coli* DH5α	*sup*E44 Δ*lac*U169 (Φ80lacZ_M15) *hsd*R17 *rec*A1*gyr*A96 *thi*-1 *rel*A1	[62]
*E. coli* FUS4	Wild type W3110 (F^−^, λ^−^ IN (*rrn*D-*rrn*E) 1, *rph*-1) ∆(*phe*A *tyr*A *aro*F) Δ*lac*::P_tac_::*aro*FBL^+^	[63]
*E. coli* FUS4.T1	Wild type W3110 (F^−^, λ^−^ IN (*rrn*D-*rrn*E) 1, *rph*-1) ∆(*phe*A *tyr*A *aro*F) Δ*lac*::P_tac_::*aro*FBL^+^ Δ*xyl*::P_tac_::*tyr*A (Y263C)	(This study)
*E. coli* FUS4.T2	Wild type W3110 (F^−^, λ^−^ IN (*rrn*D-*rrn*E) 1, *rph*-1) ∆(*phe*A *tyr*A *aro*F) Δ*lac*::P_tac_::*aro*FBL^+^ Δ*xyl*::P_tac_::*tyr*A (Y263C) ∆*tyr*R	(This study)

**Table 2:** Plasmids used in this study

Plasmid	Plasmid characteristics	References/Source
pET 20b +	P_lac_; Amp^R^; lacI^q^	Merck (Darmstadt)
pET_hpaBC	P_lac_; Amp^R^; lacI^q^	This study
pET_ddc	P_lac_; Amp^R^; lacI^q^	This study
pJNTN-m-L	P_tac_; Kan^R^; lacI^q^	[64]
pJNTN_hpaBC	P_tac_; Kan^R^; lacI^q^	This study
pJNTN_ddc	P_tac_; Kan^R^; lacI^q^	This study
pJNTN_hpaBC_ddc	P_tac_; Kan^R^; lacI^q^	This study
pTarget-sgRNA-xylAB	Cm^R^, ColE1ori	This study
pTarget-sgRNA-tyrR	Cm^R^, ColE1ori	This study
pCas	Rep101^Ts^, Km^R^	[65]
pTarget-F	Str^R^, ColE1ori	[65]
pTarget-cat	Cm^R^ gene, ColE1ori	This study
pJF119-tyrA^Tyr263Cys^	Amp^R^, ColE1ori	Unpublished

The pET plasmid system was used as a storage vector for heterologous genes, single gene insertion only (pET_hpaBC, pET_ddc). The pJNTN plasmid was used for the crude cell lysate system (single gene plasmids only: pJNTN_hpaBC, pJNTN_ddc) and plasmid library construction (bi-cistronic construct: pJNTN_hpaBC_ddc). The constructed plasmid library is available in supplementary information S3.

### Crude cell lysates system

*E. coli* FUS4.T2 pJNTN_hpaBC and pJNTN_ddc were grown overnight in 5 mL 2xTY medium containing 50 µg/mL kanamycin at 37 °C on a rotary shaker set at 130 rpm. For main cultures, 100 mL of 2xTY medium containing 50 µg/mL kanamycin was inoculated into a 500 mL baffled shaking flask to a final OD_600nm_ of 0.1 and cultivated at 37 °C on a rotary shaker set at 140 rpm. At optical density ≈ 0.6 measured at 600 nm (OD_600nm_), 1 mM IPTG, 50 µM vitamin B6, 5 mM phenylalanine, and 0.2 mM FeCl_2_ were added to the culture. After 16 h of cultivation, a final OD_600nm_ between 8 and 10 was achieved.

After cultivation, the cells were diluted to OD_600nm_ ≈ 8 using phosphate buffer for adjustments. 80 mL of the cultured broth were centrifuged at 7830 rpm for 10 min at 4 °C. The cell pellet was washed with 10 mL of phosphate buffer. After a final centrifugation at the same settings as before, the cell pellet was resuspended in 9.8 mL phosphate buffer and supplemented with 50 µL DNAse 1 (Thermofisher, Darmstadt, Germany) and 100 µL lysozyme (100 mg/mL, Sigma Aldrich, Darmstadt, Germany). Cell lysis was performed at 37 °C for 1 h on a shaker set at 800 rpm, whereas the resuspended cells were distributed in a deep well plate to allow sufficient heat transfer. After cell lysis, the cells were centrifuged at 4 °C for 10 min at 2000 rpm.

A total of 400 µL of lysate (supernatant) was mixed with 100 µL of 5 × reaction buffer in a deep well plate. The lysate was mixed in various ratios between the *E. coli* FUS4.T2 pJNTN_hpaBC lysate and the *E. coli* FUS4.T2 pJNTN_ddc lysate. The plate was sealed with a gas-permeable membrane (AeraSeal BS-25, Excel Scientific, Victorville, USA) to allow oxygen supply and was shaken for 20 h at 37 °C and 800 rpm.

### Construction of l-tyrosine production strains

The deletion of the *tyr*R repressor gene and insertion of the P_tac_-*tyr*A^Tyr263Cys^ cassette into the chromosomal DNA of the *E. coli* FUS4 strain was performed using the CRISPR-Cas system in combination with λ-Red recombineering, as described [65]. Specific pTarget-cat-sgRNA plasmids were constructed for the deletion of the *tyr*R gene and the insertion of the P_tac_-*tyr*A^Tyr263Cys^ cassette. This was done by introducing a 20-nucleotide specific guide sequence into the pTarget-cat plasmid using the inverse PCR method. Inverse PCR was performed using the Target-uni-rev primer in combination with a corresponding specific primer, sgTyrR or sgXylAB. After inverse PCR, the DNA matrix was removed by digestion with *Dpn*I and treated with T4 polynucleotide kinase. The fragments were then ligated with T4 DNA ligase. The competent cells of the *E. coli* DH5α strain were then transformed with the ligation mixture and plated on LB agar medium supplemented with chloramphenicol (25 µg/mL). From selected clones, plasmid DNA was isolated and verified using sequencing.

The strain *E. coli* FUS4.T1 was created by integrating a fragment containing a copy of the *E. coli tyr*A (Tyr263Cys) gene controlled by the P_tac_ promoter. The integration locus for the xylose metabolism operon (*xyl*A-*xyl*B) was selected. The fragment containing the P_tac_-*tyr*A ^Tyr263Cys^ cassette flanked by short homologous sequences (approximately 40–45 bp *xyl*A-*xyl*B loci) was amplified from plasmid pJF119-Ptac-tyrATyr263Cys using primers XylA-int and XylB-int. The FUS4 strain containing the pCas plasmid was transformed with the *xyl*A-P_tac_-*tyr*A^Tyr263Cys^-*xyl*B amplicon and the specific pTarget-sgRNA-xylAB plasmid by electroporation. After transformation, clones were selected on a McConkey-Agar medium containing 1% xylose, 50 µg/mL kanamycin, and 25 µg/mL chloramphenicol. The selected clones (Km^R^, Cm^R^ and Xyl^−^ phenotype) were verified using PCR (primers Xyl-scrin5' and Xyl-scrin3') and by sequencing the PCR product. Both the pCas and pTarget-sg-xylAB plasmids were removed from the resulting strain as described previously [65].

To delete the *tyr*R gene from FUS4.T1, we amplified and ligated two fragments, HR1 and HR2, which flanked the target gene. These fragments were amplified using primers tyrR-del-L5′/tyrR-del-L3′-BamH and tyrR-del-R5′-BglII/tyrR-del-R3′, respectively. After digesting HR1 with *Bam*HI and HR2 with *Bgl*II, we ligated the two fragments together and amplified them using end primers tyrR-del-L5′ and tyrR-del-R3′. The FUS4.T1 strain containing the pCas plasmid was transformed by electroporation with the resulting HR1-HR2 fragment, as well as specific pTarget-cat-sgRNA-tyrR, enabling subsequent homologous recombination by λ-red recombineering. After transformation, we selected clones on LB-Agar containing kanamycin (50 μg/mL) and chloramphenicol (25 μg/mL), and verified the resulting strain (FUS4.T2) by PCR (using primers tyrR-del-L5′ and tyrR-del-R3′) and sequencing of the PCR product. All transformation procedures were carried out using an electroporation protocol with a voltage of 2.3 kV in a 2-mm cuvette using Bio-Rad equipment.

### Construction of dopamine production strains

The genes that enable dopamine production were extracted from *E. coli* BL21 (DE3) for *hpaBC* and *Pseudomonas putida* KT2440 for *ddc*. The Monarch Genomic DNA Purification Kit (NEB, Ipswich, USA) was used to isolate the chromosomal DNA of the organisms. Genes were amplified by PCR and verified by 1% agarose gel electrophoresis. For automated DNA fragment construction by PCR, Q5 Hot Start High-Fidelity DNA Polymerase (NEB, Ipswich, USA) was used according to the manufacturer's instructions. The plasmid pJNTN-m-L was digested via *Nde*I (NEB, Ipswich, USA) according to the manufacturer's instructions with further addition of Arctic phosphatase (NEB, Ipswich, USA) and an inactivation step at 65 °C for 20 min. Fragments were then verified by 1% agarose gel electrophoresis. The different fragments were assembled by automated DNA assembly. For this purpose, PCR fragments were diluted 1:4, and 500–700 nL of each fragment mixture was combined with 75 ng of digested pJNTN-m-L or pET20b( +), 5 µL of NEBuilder HiFi DNA Assembly Master (NEB, Ipswich, USA), and water was added to reach a final volume of 10 µL. The mixture was incubated at 50 °C for 1 h.

Chemically competent cells were prepared as described previously [66]. Subsequent automated heat shock transformation was executed as described previously [66]. The transformation approach was plated on 6-well agar plates and incubated for 20 h at 37 °C. The resulting colony forming units (CFU) were analysed by colony PCR (OneTag Quick-Load 2 × Master Mix, NEB, Ipswich, USA) according to the manufacturer's instructions. CFUs with the correct length of colony PCR product were used for further plasmid isolation.

Plasmids were extracted using the NucleoSpin Plasmid Kit (Macherey–Nagel, Düren, Germany) according to the manufacturer's instructions. The isolated pJNTN_hpa_ddc, pET_hpa and pET_ddc plasmids were verified by sequencing and used for transformation. Subsequently, the *E. coli* FUS4.T2 strain was transformed with pJNTN_hpa_ddc for production experiment and *E. coli* DH5α strain with pET_hpa and pET_ddc plasmids for storage.

### Microbioreactor cultivation

Cultivation was performed using a microbioreactor system (RoboLector L, Beckman Coulter, Brea, USA) equipped with 48-well microplates (FlowerPlate, MTP-48-B, Beckman Coulter, Brea, USA), each well having a maximum volume of 1 mL and containing either 2xTY or minimal medium. Cultivation was performed under controlled conditions of 85% humidity, at a temperature of 37 °C, and with a shaking frequency of 1100 rpm. To ensure sterile conditions, each plate was sealed with a gas-permeable membrane (AeraSeal BS-25, Excel Scientific, Victorville, USA). Backscatter values were continuously monitored by the microbioreactor system.

Both precultures and main cultures were cultivated on the same plate. Wells in columns one and eight were used for the preculture, whereas the remaining columns were used for triplicate main culture experiments. The preculture was inoculated with a single CFU of *E. coli* FUS4.T2, grown in 2xTY medium, and used to inoculate the main culture. When the preculture reached a backscatter value of 12.5 (Gain 2; OD_600nm_ ≈ 4), 50 µL of the preculture broth was added to 950 µL of minimal medium for inoculation at OD_600nm_ ≈ 0.2. The main culture was subsequently cultivated in minimal medium. Unless otherwise stated, the total cultivation time was 24 h.

### Analytical methods

Quantification of l-tyrosine, l-DOPA, and dopamine was performed by analysing the cell-free supernatant of the cultivation broth. HPLC analysis was performed on an Agilent 1200 series instrument (Agilent Technologies, Santa Clara, USA) equipped with an autosampler, and a UV detector. Separation was performed on a BDS Hypersil C18 (150 × 4.6 mm, 5 μm; Thermo Scientific: 28105-154630) which was protected by an BDS Hypersil C18 Drop-in (10 × 4 mm, 5 μm; Thermo Scientific: 28105-014001). Fluorometric detection (excitation at 230 nm and emission at 280 nm) was performed. The degassed mobile phase consisted of 0.2% trifluroacetic acid and 10% [v/v] methanol in water. The column was heated to 30 °C, the flow rate was set to 0.4 mL/min and the injection volume was 10 µL. Standards were measured over a range of 0.1 mM to 5 mM.

If necessary, optical density of the cell culture was measured at a wavelength of 600 nm. Glucose concentrations were quantified using a LaboTrace automatic analyser (TraceAnalytics GmbH). LC–MS/MS proteomics was performed externally by the Hohenheim Core Facility.

## Results

### Host strain engineering leads to high l-tyrosine supply

The precursor for dopamine synthesis is l-tyrosine. Consequently, a l-tyrosine-producing strain was engineered to ensure sufficient l-tyrosine supply for the subsequent formation of dopamine. The strain *E. coli* FUS4 of Gottlieb et al. is auxotrophic for l-phenylalanine and l-tyrosine and served as a parental strain [63]. Subsequent strain engineering focused on the integration of the modified gene *tyrA,* and the deletion of the gene encoding the TyrR regulator (*tyrR*) (Fig. 1). The TyrA protein was modified at residue 263 (Y263C) to overcome feedback inhibition [23], which allows accumulation of high l-tyrosine concentrations in the medium. In the FUS4.T1 strain, only *tyrA* was overexpressed and mutated, whereas the additional deletion of *tyrR* resulted in a 38% increase in l-tyrosine concentration of FUS4.T2. Table 3 shows that the relative transcription increase of *tyrA* corresponds with the increasing titre. To note, FUS4.T2 remains l-phenylalanine auxotrophic.

**Table 3:** Summary of the key characteristics of different l-tyrosine production strains including the strains constructed in this study

Strain	Genotype	Rel. transcription *tyrA*	l-Tyrosine [g/L]	Resource
*E. coli* K12 ΔtyrR	K12 ΔtyrR pCL1920::_PLtetO−1_aroG^fbr^tyrA^fbr^ppsAtktA^b^	–	0.62 ± 0.026	[67]
F	E. coli MG1655 pBbA5a::tyrB-tyrA*-aroC T1-P_*trc*_-aroA-aroL pBbB5c::aroE-aroD-aroBop-aroG*-ppsA-tktA	–	2.17 ± 0.38	[68]
SCK5	W3110 Δ*tyrR aroG*:: P_BBa_J23100_-synUTR_aroG_-*aroG*^*fbr*^* tyrA*:: P_BBa_J23100_-synUTR_tyrA_-*tyrA*^*fbr*^ P_aroABCDELtyrB_-UTR_aroABCDELtyrB_:: P_BBa_J23100_-synUTR_aroABCDELtyrB_ P_ppsA_-UTR_ppsA_:: P_BBa_J23100_-synUTR_ppsA(V4)_	–	0.52 ± 0.17	[69]
FUS4	LJ110 ∆(*phe*A *tyr*A *aro*F) Δ*lac*::P_tac_::*aro*FBL^+^	0.05 ± 0.01	–	[63]
FUS4.T1	LJ110 Δ(∆(*phe*A *tyr*A *aro*F) Δ*lac*::P_tac_::*aro*FBL^+^ Δ*xyl*::P_tac_::*tyr*A (Y263C)	30.59 ± 1.20	0.60 ± 0.02	This study
FUS4.T2	LJ110 ∆(*phe*A *tyr*A *aro*F) Δ*lac*::P_tac_::*aro*FBL^+^ Δ*xyl*::P_tac_::*tyr*A (Y263C) ∆*tyr*R	48.01 ± 1.32	0.83 ± 0.01	This study

The strains *E. coli* FUS4, *E. coli* FUS4.T1, and *E. coli* FUS4.T2 were investigated in relative transcription of *tyrA* (*n* = 3) and l-tyrosine production. Relative expression was calculated according to the housekeeping gene *ftsZ*. l-tyrosine concentration was measured after 52 h of cultivation in a microbioreactor device (*n* = 3)

The majority of l-tyrosine is produced during the initial growth phase (supplementary information S4). Hence, *E. coli* FUS4.T2 was qualified as an optimal host for the production of tyrosine-derived products. No further adjustments were made to improve l-tyrosine production, as the primary focus of this work was to optimise dopamine formation.

**Fig. 2:**
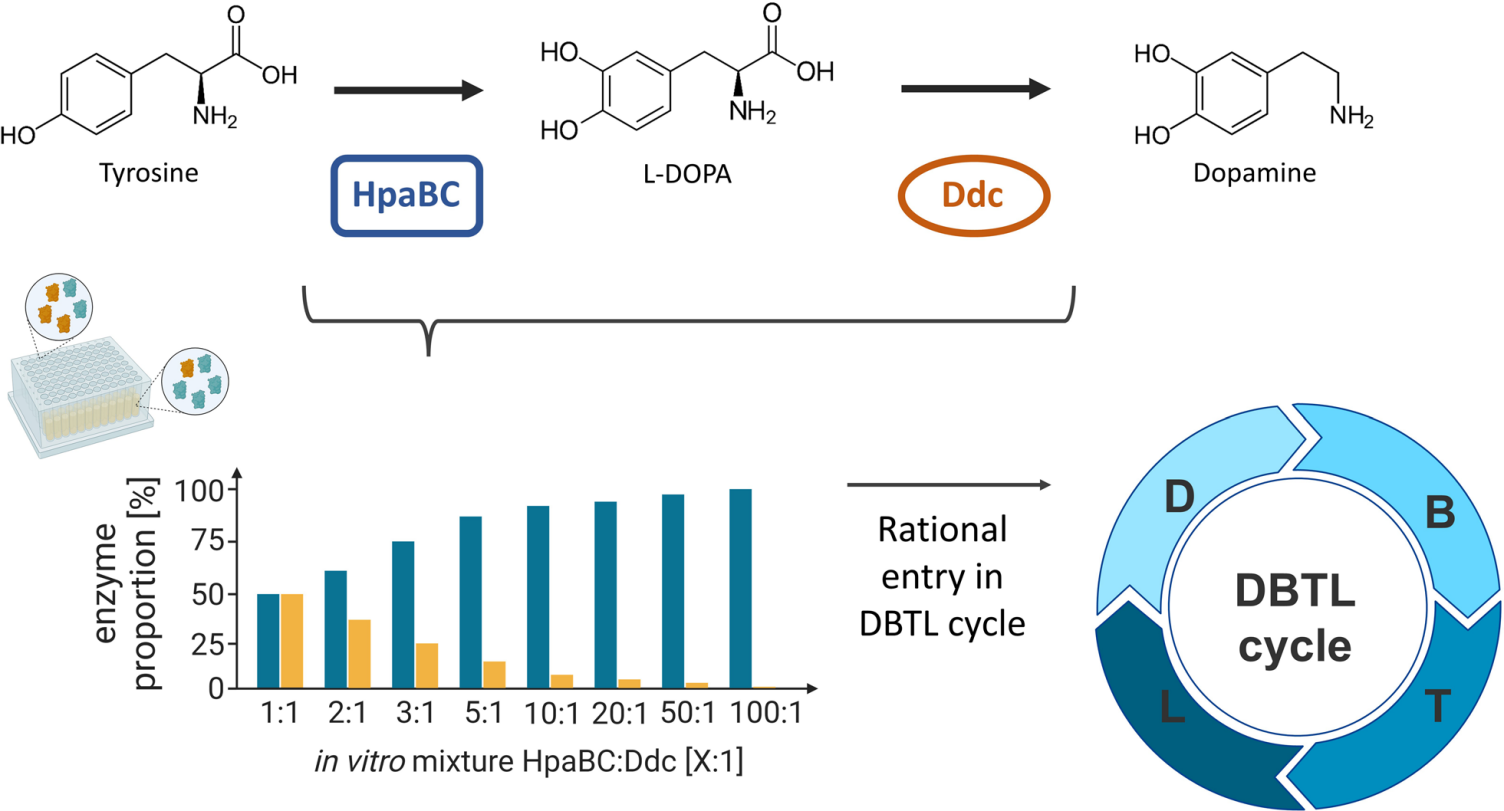
The knowledge driven DBTL cycle for rational dopamine strain engineering. In the upper part, the schematic pathway for dopamine production based on l-tyrosine via l-DOPA to dopamine is shown. The enzymes 4-hydroxyphenylacetate 3-monooxygenase (HpaBC) and l-DOPA decarboxylase (Ddc) are responsible for the respective conversions. The rational design of the pathway is performed by preliminary in vitro experiments. Based on the optimal ratio of the pathway components, the DBTL cycle can be entered in a rational way. Therefore, effective and straightforward strain engineering is possible. Partly created in BioRender. Takors (2025) https://biorender.com/m17m852

In order to establish dopamine production, it is essential to overexpress two biosynthetic genes: *hpaBC,* encoding the 4-hydroxyphenylacetate 3-monooxygenase, and *ddc,* encoding the l-DOPA decarboxylase (Fig. 2). These genes were amplified by PCR from the genome of *E. coli* BL21 (DE3) and *P. putida* KT2440, respectively.

### Concept of the knowledge driven DBTL cycle for efficient engineering of a dopamine production strain

Often, rather randomised and statistical methods are applied to enter the DBTL cycle [24, 39–42]. Here, we follow a rational approach by using the crude cell lysate method [44, 49], in order to implement the robotic DBTL workflow (supplementary information S5) using *E. coli* (Fig. 2). Fundamental enzyme kinetics were investigated as a prerequisite for the subsequent knowledge driven optimisation. The in vitro enzyme kinetic study builds upon the approaches of Dudley et al. and Karim et al. [43, 44], enabling more efficient fine-tuning pathways. Accordingly, crude cell lysates of two single enzyme-producing strains were mixed, enabling the analysis of in vitro product formation. This technique provides key insights for optimal DBTL design, reduces DBTL iterations, and enhances process efficiency.

Following in vitro investigations, the conventional DBTL cycle was applied to further fine-tune dopamine production. To accomplish this, high-throughput RBS engineering was implemented, as this is a suitable method for graduated tuning of gene expression [51–53]. The production strains were designed in silico (primer containing an engineered optimal RBS), amplified via PCR, and integrated into the pJNTN-m-L backbone vector through scarless Gibson assembly. The *E. coli* DH5α was transformed with the reaction mixture, followed by strain selection and quality control. Once the correctly assembled plasmids were isolated, the production strain (*E. coli* FUS4.T2 pJNTN_hpa_ddc) was constructed via heat shock transformation. Testing was executed in a high throughput microbioreactor system, with subsequent offline analysis of product and biomass concentration. The evaluation of this automated DBTL workflow was conducted through analysis of the success rate [70], which is defined as the percentage of correctly constructed strains in relation to the designed strains.

The insights gained from the initial knowledge driven DBTL cycle will inform a subsequent RBS engineering strategy, with the objective to achieve enhanced product titres and to identify the most efficient producer strain.

### In vitro rational dopamine pathway design enables systematic in vivo design

The de novo design of metabolic pathways relies on the known properties of involved enzymes. Regarding the dopamine pathway, the enzyme parameters of Ddc are known [16], whereas HpaBC is not completely characterised [15]. Hence, in vitro studies expressed *hpaBC* and *ddc* genes separately, followed by mixing the lysates, similar to the methods described by Dudley et al. [43] and Karim et al. [44].

**Fig. 3:**
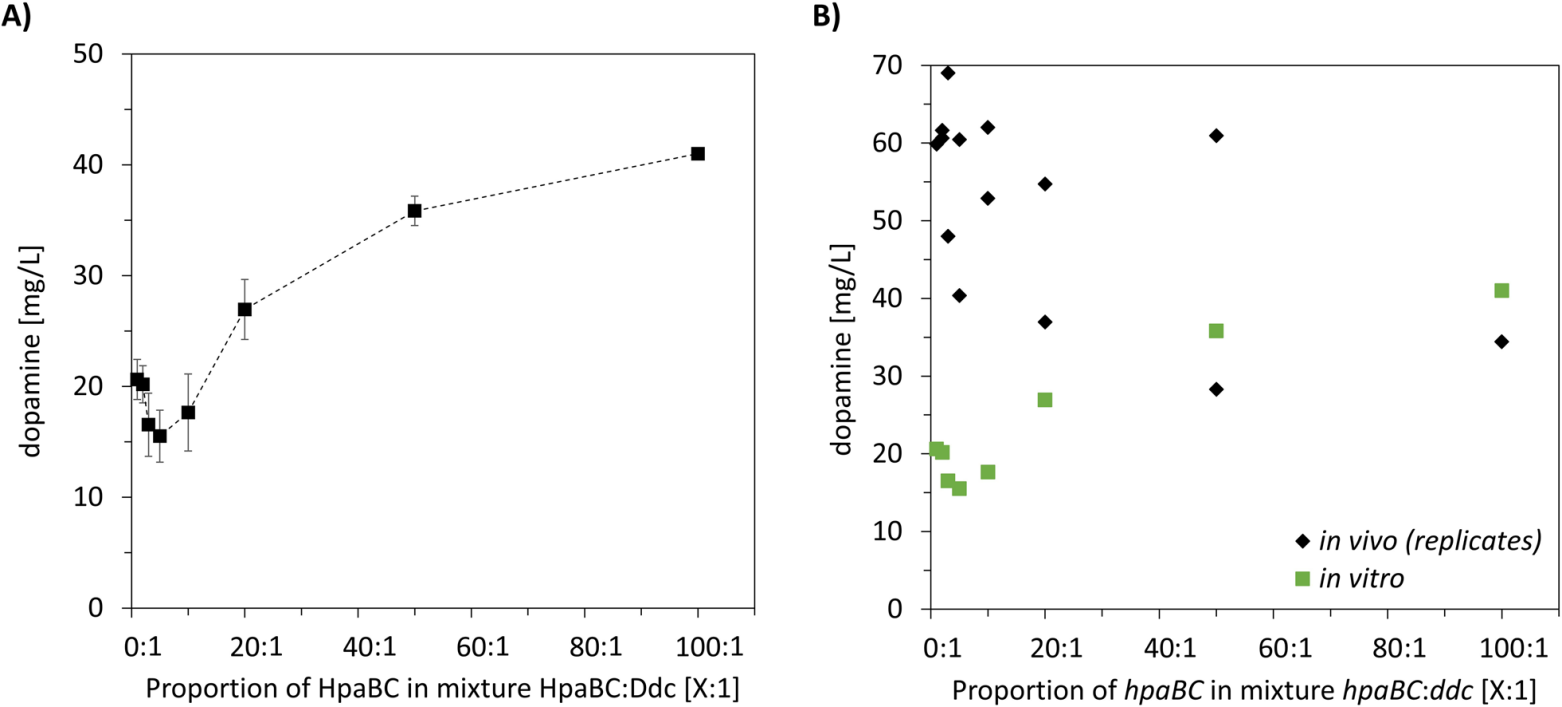
In vitro and in vivo investigation of dopamine production in *E. coli* FUS4.T2. **A** Supernatants of lysed cells were mixed in different ratios with increasing amounts of HpaBC. Data represent mean of replicates (*n* = 3) with standard deviation as error bars. **B** For in vivo investigation of different *hpaBC:ddc* ratio duplicates were made using different RBS sequences (black diamond) compared with the in vitro results (green square). HpaBC: 4-hydroxyphenylacetate 3-monooxygenase; Ddc: l-DOPA decarboxylase; TIR: translation initiation rate; RBS: ribosome binding site

The K_M_ value of Ddc has been reported as 0.092 ± 0.019 mM for l-DOPA [15, 16]. By contrast, the K_M_ of HpaBC ranges between 0.0094 ± 0.0016 mM for 4-hydroxyphenylacetate and 0.514 ± 0.1 mM for methyl hydroxybenzoate, both of which are structurally different from l-tyrosine, the targeted substrate of this study. As accumulation of l-DOPA has the potential to promote spontaneous oxidation and thus melanin production, balanced enzyme levels are required to achieve efficient conversion of l-tyrosine to dopamine without accumulating intermediates and reduced product formation. Hypothesizing that Ddc is catalytically more efficient than HpaBC, we investigated the effect of increasing HpaBC to Ddc ratios, ranging from 1:1 to 100:1. Increasing the ratio of HpaBC to Ddc resulted in progressively higher dopamine titres, achieving an overall increase of 98% (Fig. 3A). Given the converging trend we concluded that much higher amounts of HpaBC are needed to equilibrate the flux to dopamine.

### High-throughput design for RBS engineering

Next, we aimed to effectively translate the in vitro results into the in vivo dopamine pathway design by engineering the Shine-Dalgarno (SD) sequence regions of *hpaBC* and *ddc*. This approach should enable the fine-tuning of gene expression, which is crucial for further mechanistic strain improvement.

**Fig. 4:**
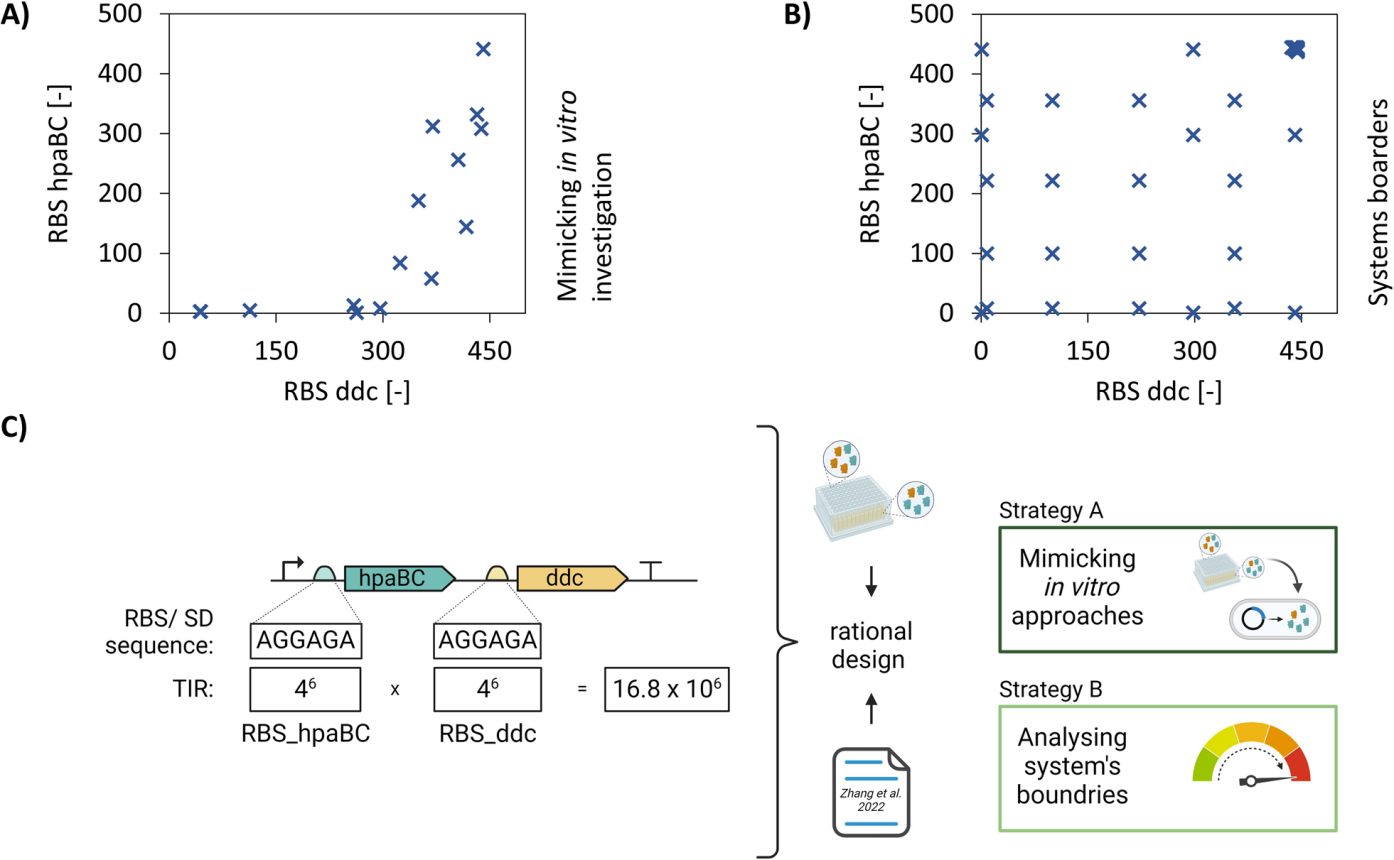
High-throughput ribosome binding site (RBS) engineering for optimised dopamine production. Number of RBS relates to the already tested RBS from Zhang et al. [57] **A** Overview of tested RBS combinations for mimicking the in vitro investigation (Strategy A). Two compositions with the highest degree of similarity were selected for each in vitro analysis point. **B** A random selection of independent points was made in order to analyse the system boundary (Strategy B). **C** Principle of RBS engineering using the 6 base pair Shine-Dalgarno (SD) sequence as mutation site. Up to 16.8 million combinations (4^6^ × 4^6^) in the bi-cistronic context are possible. Rational design was therefore applied using in vitro data as well as literature data from Zhang et al. [57]. The two different key strategies for RBS engineering were applied. Created in BioRender. Takors (2025) https://biorender.com/t91y551

The SD sequence, composed of six nucleotides (AGGAGA), theoretically offers 4,096 possible combinations (4^6^) for a single sequence, and up to 16.8 million combinations (4^6^ × 4^6^) in the bi-cistronic context. Previous studies have characterised a subset of RBS sequences by engineering the SD region in order to better understand its function and develop reliable design methods [57]. As key performance indicators, TIRs were calculated by measuring GFP expression and biomass accumulation. TIR, as characteristic pattern for RBS sequences, provided the starting point for designing bi-cistronic expression systems, allowing precise fine-tuning of relative enzyme expression levels (Fig. 4C).

Two complementing strategies were followed to screen the parameter space:

- Strategy A: Mimicking in vitro approaches within the in vivo environment
- Strategy B: Analysing the system's boundaries to identify potential limitations and opportunities

Hence, ‘A’ checks the transferability of the in vitro results whereas ‘B’ aims to identify application boundaries.

Strategy A assumes that the TIR ratio of two subsequent genes in one operon basically determines the ratio of the translated protein products. For simplicity, the findings of the in vitro tests were translated to in vivo application by identifying RBS pairs of *hpaBC* and *ddc* that show the same TIR ratio as in related in vitro tests. This assumption is limited, since it does not consider dilution factors that affect in vivo tRNA availability, as well as post translational and folding regulations. However, TIRs of all RBS sequences were calculated. Next, TIR(hpaBC) was multiplied by TIR(ddc) to identify appropriate couples matching the numerical ratio within the in vitro tests. For instance, if the in vitro ratio was 2:1 (resulting in a numerical ratio of 0.5), a corresponding in vivo overall TIR ratio of 0.5 was established through RBS engineering. The intention was to test not only single RBS combinations but to check two fitting RBS combinations at least. In total, 15 different combinations were selected for detailed analysis (see Fig. 4A).

To further expand the design space and enable the mechanistic analysis of the dopamine biosynthesis pathway (Strategy B), RBS pairs were handpicked from the pool of characterised RBS sequences [57] and further complemented with the strongest RBS candidates. Tests with the SD sequence ‘AGGAGA’ in both genes served as reference in all experimental series. Summarizing, strategy B comprised 41 different combinations (Fig. 4B).

The sum of RBS designs (15 + 41 = 56) were implemented into *E. coli* FUS4.T2 using the bi-cistronic vector pJNTN_hpaBC_ddc (supplementary information S3) applying the automated strain construction workflow (supplementary information S5). Finally, 51 out of 56 designs could be successfully constructed and further analysed, indicating a success rate of 91.1%.

### Mechanistic understanding of heterologous dopamine pathway design

**Fig. 5:**
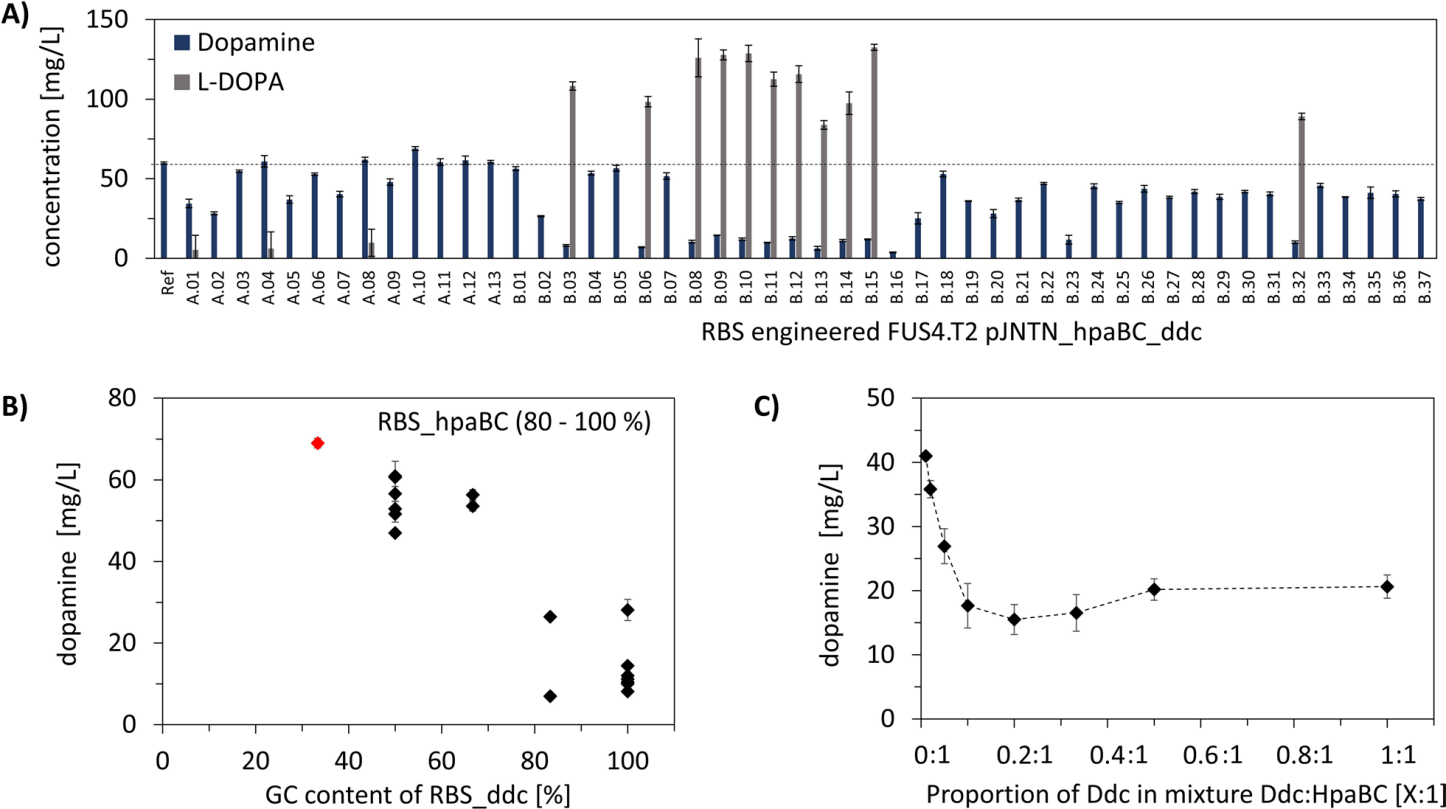
Mechanistic understanding of heterologous dopamine pathway engineering. **A** Production of dopamine (blue bars) and l-DOPA (grey bars) by RBS engineered *E. coli* FUS4.T2 pJNTN_hpaBC_ddc. **B** The GC content of RBS_hpaBC was kept at 80–100% and different GC content of RBS_ddc was analysed. The best producer was marked in red. **C** In vitro rational pathway engineering was applied for dopamine synthesis. The supernatants from lysed cells were combined in varying ratios with increasing concentrations of Ddc. Data represent mean of replicates (*n* = 3) with standard deviation as error bars

Values A.01-A.13 of Fig. 5A represent the results of the in vitro to in vivo transfer according to strategy A. Apparently, trends of the in vitro tests could not be reproduced in vivo. Initial evaluation of the design to mimic the in vitro to in vivo transitions, revealed that the intended ratios of RBS_hpaBC to RBS_ddc did not produce identical results in both environments (Fig. 3). Two in vivo strains were designed to mimic one in vitro ratio, and constructs with stronger RBS_hpaBC (high TIR ratio) consistently produced higher dopamine titres (Fig. 3B, supplementary information S6). However, strain A.10 yielded the highest dopamine titre (69.03 ± 1.2 mg/L), an increase of 15% compared to the reference.

Surprisingly, results applying strategy B (Fig. 5A: B.01-B.37) revealed constructs with low dopamine but high l-DOPA titres (e.g. B.03, B.11, B.32). Additional proteomics indicated reduced Ddc contents in strains B.03 and B.11, despite the use of (hypothesized) strong RBS sequences (supplementary information S7).

The poor predictability of TIR based dopamine production motivated us to consider the impact of GC content in RBS as suggested by Zhang et al. [57] and Kuo et al. [71]. The GC content of the reference strain was 50% for both RBS sequences. Correlating the RBS GC content with the achieved dopamine titres revealed that dopamine production decreased when the GC content remained at 100% in RBS_hpaBC while the GC content in RBS_ddc increased. Accordingly, low GC in RBS_ddc amplified dopamine production (Fig. 5B). Conversely, maintaining 100% GC content for RBS_ddc while reducing GC content in RBS_hpaBC decreases dopamine production (supplementary information S6). Consequently, highest dopamine titres were achieved with high and low GC contents in RBS_hpaBC and RBS_ddc, respectively.

Summarizing, the GC content of RBS turns out to be the key indicator of RBS strength and translation efficiency. Comparing in vitro and in vivo outcomes confirmed that high GC content in RBS_hpaBC (80–100%) and reduced GC content in RBS_ddc optimize dopamine production, as shown with the top-performing strain A.10 (69.03 ± 1.2 mg/L) (Fig. 5B). These results indicate that increasing the HpaBC:Ddc ratio, either in vivo or in vitro, increases dopamine production (Fig. 5B and C).

LC–MS/MS Proteomic analysis demonstrated that increasing RBS_hpaBC GC content not only improved *hpaBC* expression but also the formation of the related protein. The opposite observation was made regarding Ddc: Increasing RBS_ddc GC content reduced Ddc levels whereas decreasing the GC content of RBS_ddc lead to increased *ddc* expression (supplementary information S7) and likewise elevated Ddc protein levels. Apparently, the optimum GC content for RBS is gene context dependent. In the particular application, dopamine production can be enhanced by reducing RBS_ddc GC content and maximizing RBS_hpaBC GC content.

Summarizing, this study successfully developed a dopamine production strain making use of the knowledge driven DBTL cycle. Key findings from the in vitro analysis drove in vivo studies, ultimately identifying pinpointing to RBS engineering as the most impactful tool for balancing active enzyme levels in order to optimise production.

## Discussion

This study demonstrates the development and optimisation of a dopamine production strain by implementing the knowledge driven DBTL cycle for rational strain engineering. This was done using a combination of in vitro investigation with in vivo pathway optimisations, employing high-throughput RBS engineering enhances dopamine production.

To develop and optimise a dopamine production strain, it was crucial to establish a host strain platform that would facilitate dopamine production by providing the essential precursor, l-tyrosine. Strategic host strain modifications were made to provide a platform for subsequent engineering. This resulted in enhanced l-tyrosine production as a precursor for dopamine production. The *E. coli* FUS4 strain was used as the parental strain and modified by deletion of *tyrR* and overexpression of engineered *tyrA*, resulting in the *E. coli* FUS4.T2 strain. Table 3 indicates that *E. coli* FUS4.T2 produces an average amount of l-tyrosine (0.83 ± 0.01 g/L), aligning well with other studies [67–69]. In contrast, only engineering and overexpression of *tyrA* showed lower l-tyrosine production (0.60 ± 0.02 g/L). The objective of this study was not to develop an optimised l-tyrosine production strain, but rather to establish a fundamental l-tyrosine producer as the starting point for dopamine production. It should be noted that there are additional genome engineering points to consider such as inactivating the phosphotransferase system [72] by increasing the *ppsA* gene [73, 74]. However, based on the established host strain *E. coli* FUS4.T2, pathways for l-tyrosine derived products can be implemented into the strain.

The development of the dopamine producer used the knowledge driven DBTL cycle, starting with in vitro cell lysate studies to gain a mechanistic understanding of the heterologous biosynthesis. In agreement with others [43, 44, 75] the crude cell lysate approach proved to be easy-to-apply. However, this advantage may be scrutinised by comparing alternate in vitro methods that offer higher precision [76, 77]. In this study, individual enzyme levels implemented as HpaBC to Ddc ratios ranging from 1:1 to 100:1 were investigated to study enzymatic interactions. In accordance with other studies [43, 44, 75], the design space was efficiently screened, also providing a mechanistic understanding of the interacting partners that is not provided by alternate randomised designs [39, 41, 42, 78, 79].

Thanks to the preliminary in vitro study, the DBTL cycle could be entered with an enhanced mechanistic understanding. The toolbox of gene expression modulation enables the engineering of various gene regulation modules, promoters, RBS, and start codons [50]. In this study, RBS engineering merged as a promising tool to fine tune gene expression and protein levels [51–53, 57].

In order to ensure the optimal and reliable functioning of the bi-cistronic system, the following conditions have been installed to enable systematic RBS engineering: the *ddc* gene was placed after the *hpaBC* gene to ensure that *hpaBC* has the highest expression [80, 81], and the stop codon was swapped for the strong stop codon (TAG) to prevent read through [82]. Several studies have previously analysed polycistronic operons, albeit with a relatively low throughput. One study employed machine learning for this purpose, yet no mechanistic understanding was integrated [59]. Another study applied RBS engineering to enhance violacein production, achieving a 2.41 fold increase in titre [60]. Höllerer et al. employed a large high-throughput approach integrating deep learning, systematically testing RBS sequences and their surrounding context. This study revealed that the 17 bases upstream of the start codon are critical for efficient translation [83]. The RBS engineering approach, within the knowledge driven DBTL cycle, addresses the first 17 bases upstream of the start codon.

By fine-tuning dopamine production using RBS engineering, final dopamine titres increased by 15.3%, reaching 69.03 ± 1.2 mg/L, equivalent to 34.34 ± 0.59 mg/g_biomass_. Notably, the best producer resulted from strategy A, which successfully mimicked the in vitro approach in the in vivo environment. Compared to the benchmark set by Das et al. [21], which reported reaching dopamine titres of 27 mg/L and a product-per-biomass yield of 5.17 mg/g_biomass_, our approach improved performance by 2.6 and 6.6-fold, respectively. However, l-DOPA accumulation was observed in strains A.01, A.04, A.08, B.03, B.06, B.08-B.15, B.32 indicating insufficient Ddc levels. Apparently, the active enzyme levels of HpaBC and Ddc require well harmonized balancing for optimum production, with the ideal ratio determined to be 2.6: 1 in this study. Prospective research with enhanced l-tyrosine supply may determine whether this ratio remains optimal, or needs to be adjusted to support even higher carbon fluxes through the biosynthetic pathway.

The investigations clearly demonstrate the impact of GC content in the SD sequence on the RBS strength and on the level of gene expression. For *hpaBC* expression, our findings are consistent with previous studies [57, 71]. Higher GC content correlates with a stronger RBS, whereas lower GC content is associated with a weaker RBS. This relationship can be attributed to the increased number of hydrogen bonds between the GC base pairs. However, for *ddc* expression, the opposite could be shown. This suggests that the strength of the RBS is dependent on the gene context [51, 81, 84]. Another mechanism associated with bi-cistronic expression is translational coupling [85, 86]. Levin-Karp et al. demonstrated an increase in gene expression of the first gene when gene expression of the second gene was enhanced [81], but this is not the case in our study. However, Ddc protein levels are quite low compared to HpaBC protein levels. This could be due to the bi-cistronic architecture as described above [80, 81], but also due to secondary structures within the bi-cistronic system [87, 88]. Assuming that GC content is responsible for RBS strength, we again compared the in vitro data with the high-throughput data. Our results demonstrated that both approaches result in reduced *ddc* expression, coupled with increased *hpaBC* expression, which in turn leads to enhanced dopamine production. However, this finding does not consider dilution factors within the in vivo protein expression.

To achieve these results, it was necessary to add automation to the knowledge driven DBTL cycle. In the following, we will evaluate the holistic automation workflow. The existing automated DBTL workflow was employed in order to address the two design questions that had been defined (mimicking in vitro approaches within the in vivo environment and analysing the system's boundaries to identify potential limitations and opportunities). Out of the 56 constructs that were created, 51 strains were successfully constructed, and 48 strains were successfully sequenced. Limitations of the workflow were successful PCR reaction (1 strain), DNA assembly and transformation (4 strains), and point mutations in the RBS (3 strains). This places the success rate of the high-throughput DBTL workflow at 85.7% to 91.1%. In comparison, other approaches show success rates of 37.5% (semi-automated, Tenhaef et al. [89]) to 96.2% (fully automated, Chao et al. [90]). Nava et al. reported success rates of 13.6% for positively sequenced strains and 20.6% for positively constructed strains [70], while Rosch et al. observed a 56.3% success rate for positively screened strains [91]. This aligns the success rate of our workflow within the range of other approaches.

In this work, we were able to develop and optimise an efficient dopamine production strain by implementing the knowledge driven DBTL cycle involving upstream in vitro investigation. In the subsequent round of the knowledge driven DBTL cycle, the dopamine production could be further increased by implementing the knowledge gained. Incorporating machine learning tools promises to advance automation even further by identifying optimal candidates for the next DBTL iteration. The platform demonstrated here not only streamlines strain construction within the bi-cistronic framework but is also adaptable to produce a variety of target molecules.

## Conclusions

The aim of this work was to optimise dopamine production in *E. coli*. This was achieved by implementing the knowledge driven DBTL cycle, starting with upstream in vitro investigation. This automated workflow facilitates both mechanistic understanding and efficient DBTL cycling. In vitro investigation demonstrated that the active enzyme levels of HpaBC and Ddc require well harmonized balancing for optimum dopamine production. By applying RBS engineering within the knowledge driven DBTL cycle, we developed a strain capable of producing dopamine at concentrations of 69.03 ± 1.2 mg/L which equals 34.34 ± 0.59 mg/g_biomass_. Thereby, we clearly highlighted how the GC content in the SD sequence influences the strength of the RBS and the level of gene expression. Key findings from these studies pinpoint RBS engineering as the most effective tool for balancing active enzyme levels to optimise dopamine production.

## Supplementary Information

Supplementary Material 1: S1. Primer used in this study. S2. DNA sequences of the extracted genes (*hpaBC*, *ddc*). S3: Library of different high throughput constructs and the different RBS sequences of *hpaBC* and *ddc. *S4. Host strain construction of *E. coli* FUS4.T1 and FUS4.T2. S5. Overview of the semi-automated DBTL workflow. S6. Evaluation of different RBS combinations. S7: Proteomics data.

## Data Availability

The datasets supporting the conclusions of this article are available in the DaRUS repository [10.18419/darus-4714].

## Abbreviations

CFU: Colony forming units
OD: Optical density
Ddc: L-DOPA decarboxylase
HpaBC: 4-Hydroxyphenylacetate 3-monooxygenase
TIR: Translation initiation rate
*E. coli*: *Escherichia coli*
CFPS: Cell-free protein synthesis
RBS: Ribosome binding site
SD: Shine-Dalgarno
DBTL cycle: Design-built-test-learn cycle
