# Supplementary material for: The knowledge driven DBTL cycle provides mechanistic insights while optimising dopamine production in Escherichia coli
Source: Microb Cell Fact. 2025 May 16;24:111. doi: 10.1186/s12934-025-02729-6 (PMC12084978; doi:10.1186/s12934-025-02729-6)
Supplement: Supplementary file 1 — Supplementary Material 1: S1. Primer used in this study. S2. DNA sequences of the extracted genes (hpaBC, ddc). S3: Library of different high throughput constructs and the different RBS sequences of hpaBC and ddc. S4. Host strain construction of E. coli FUS4.T1 and FUS4.T2. S5. Overview of the semi-automated DBTL workflow. S6. Evaluation of different RBS combinations. S7: Proteomics data. [file 12934_2025_2729_MOESM1_ESM.pdf]

Supplementary Information

The following information is provided to the article:

The knowledge driven DBTL cycle provides mechanistic insights while optimising dopamine production in *Escherichia coli*

Lorena Hägele[1], Natalia Trachtmann[1,2] and Ralf Takors[1]*

[1]Institute of Biochemical Engineering, University of Stuttgart, Stuttgart 70569, Germany

[2]Laboratory of Molecular Genetics and Microbiology Methods, Kazan Scientific Center of Russian Academy of Sciences, 420111 Kazan, Russia

***Correspondence:** Ralf Takors, Institute of Biochemical Engineering, University of Stuttgart, Allmandring 31, Stuttgart 70569, Germany. Email: ralf.takors@ibvt.uni-stuttgart.de

Supplementary Information S1:

Primers used in this study are listed in Supplementary Table 1.

Table 1: Primer used in this study. All primers were used for Gibson assembly, with the exception of genome extraction primer, sequencing primer and quality check primer. HT primer have the engineered RBS sequence included. Abbreviations: HT: high-throughput

No.	Oligonucleotide	Sequence (5'-> 3')	function
1	ext_ddc_fwd	gtgacccccgaacaattccg	Genome extraction
2	ext_ddc_rev	tcagcccttgatcacgtcc	Genome extraction
3	ext_hpaBC_fwd	atgaaaccagaagatttccgcgccag	Genome extraction
4	ext_hpaBC_rev	ttaaatcgcagcttccatttccagcatcac	Genome extraction
5	pET_fwd	attgcactcgagcaccaccacc	Storage vector backbone
6	pET_rev	atgtatatctccttcttaaagttaaacaaaattatttc	Storage vector backbone
7	ddc_pET_fwd	ctttaagaaggagatatacatatgacccccgaacaattcc	Storage vector ddc
8	ddc_pET_rev	ggtggtgctcgagtgcaatgcccttgatcacgtcctg	Storage vector ddc
9	hpaBC_pET_fwd	ctttaagaaggagatatacatatgaaaccagaagatttccgcgccag	Storage vector hpaBC
10	hpaBC_pET_rev	ggtggtgctcgagtgcaatcgcagcttccatttccagc	Storage vector hpaBC
11	ddc_pJN_1_fwd	caatttcacacaggagatatacaatgacccccgaacaattcc	*in vitro* vector ddc
12	ddc_pJN_1_rev	ccccgggtaccgagctcgacatcagcccttgatcacgtcctg	*in vitro* vector ddc
13	hpa_pJN_1_fwd	caatttcacacaggagatatacaatgaaaccagaagatttccgcg	*in vitro* vector hpaBC
14	hpa_pJN_1_rev	caatgcccagaaggtgttgcgc	*in vitro* vector hpaBC
15	hpa_pJN_2_fwd	cgcaacaccttctgggcattg	*in vitro* vector hpaBC
16	hpa_pJN_2_rev	ccccgggtaccgagctcgacattatcaaatcgcagcttccatttccag	*in vitro* vector hpaBC
17	hpa1_fwd_1	tgtgagcggataacaatttccattgtttaactttaagaggttggtatacatatg	HT RBS_hpaBC
18	hpa1_fwd_2	tgtgagcggataacaatttccattgtttaactttaagagcgtactatacatatg	HT RBS_hpaBC
19	hpa1_fwd_3	tgtgagcggataacaatttccattgtttaactttaagagctcattatacatatg	HT RBS_hpaBC
20	hpa1_fwd_4	tgtgagcggataacaatttccattgtttaactttaagactcggatatacatatg	HT RBS_hpaBC
21	hpa1_fwd_5	tgtgagcggataacaatttccattgtttaactttaagacgggagtatacatatg	HT RBS_hpaBC
22	hpa1_fwd_6	tgtgagcggataacaatttccattgtttaactttaagacatcagtatacatatg	HT RBS_hpaBC
23	hpa1_fwd_7	tgtgagcggataacaatttccattgtttaactttaagaaggggttatacatatg	HT RBS_hpaBC
24	hpa1_fwd_8	tgtgagcggataacaatttccattgtttaactttaagacgctattatacatatg	HT RBS_hpaBC
25	hpa1_fwd_9	tgtgagcggataacaatttccattgtttaactttaagataggactatacatatg	HT RBS_hpaBC
26	hpa1_fwd_10	tgtgagcggataacaatttccattgtttaactttaagatggcattatacatatg	HT RBS_hpaBC
27	hpa1_fwd_11	tgtgagcggataacaatttccattgtttaactttaagaggcaggtatacatatg	HT RBS_hpaBC
28	hpa1_fwd_12	tgtgagcggataacaatttccattgtttaactttaagataggagtatacatatg	HT RBS_hpaBC
29	hpa1_fwd_13	tgtgagcggataacaatttccattgtttaactttaagaaagaggtatacatatg	HT RBS_hpaBC
30	hpa1_fwd_14	tgtgagcggataacaatttccattgtttaactttaagaggcgactatacatatg	HT RBS_hpaBC
31	hpa1_fwd_15	tgtgagcggataacaatttccattgtttaactttaagaaggagatatacatatg	HT RBS_hpaBC
32	hpa1_fwd_16	tgtgagcggataacaatttccattgtttaactttaagacccgaatatacatatg	HT RBS_hpaBC
33	hpa1_fwd_17	tgtgagcggataacaatttccattgtttaactttaagagcttggtatacatatg	HT RBS_hpaBC
34	hpa1_fwd_18	tgtgagcggataacaatttccattgtttaactttaagatgcttatatacatatg	HT RBS_hpaBC
35	hpa1_fwd_19	tgtgagcggataacaatttccattgtttaactttaagatggtattatacatatg	HT RBS_hpaBC
36	hpa1_fwd_20	tgtgagcggataacaatttccattgtttaactttaagagggggctatacatatg	HT RBS_hpaBC
37	hpa1_fwd_21	tgtgagcggataacaatttccattgtttaactttaagaggcgagtatacatatg	HT RBS_hpaBC

38	hpa1_fwd_22	tgtgagcggataacaatttccattgtttaactttaagacaggagtatacatatg	HT RBS_hpaBC
39	hpa1_fwd_23	tgtgagcggataacaatttccattgtttaactttaagaggggggtatacatatg	HT RBS_hpaBC
40	hpa1_fwd_24	tgtgagcggataacaatttccattgtttaactttaagaggcgtttatacatatg	HT RBS_hpaBC
41	hpa1_fwd_25	tgtgagcggataacaatttccattgtttaactttaagagggggttatacatatg	HT RBS_hpaBC
42	hpa1_fwd_26	tgtgagcggataacaatttccattgtttaactttaagaggctattatacatatg	HT RBS_hpaBC
43	hpa1_fwd_27	tgtgagcggataacaatttccattgtttaactttaagaaggagatatacatatg	HT RBS_hpaBC
44	hpa1_fwd_28	tgtgagcggataacaatttccattgtttaactttaagaggggcgtatacatatg	HT RBS_hpaBC
45	hpa1_fwd_29	tgtgagcggataacaatttccattgtttaactttaagaacttcctatacatatg	HT RBS_hpaBC
46	hpa1_rev	caatgcccagaaggtgttg	HT RBS_hpaBC
47	hpa2_fwd	cgcaacaccttctgggcattg	HT RBS_ddc
48	hpa2_rev	gccagttgtttaccctcacaacctaaatcgcagcttccatttccag	HT RBS_ddc
49	ddc_fwd_1	gttgtgagggtaaacaactggcgtttaactttaagatagtcgtatacatatg	HT RBS_ddc
50	ddc_fwd_2	gttgtgagggtaaacaactggcgtttaactttaagaaaatactatacatatg	HT RBS_ddc
51	ddc_fwd_3	gttgtgagggtaaacaactggcgtttaactttaagagttgagtatacatatg	HT RBS_ddc
52	ddc_fwd_4	gttgtgagggtaaacaactggcgtttaactttaagaacttcctatacatatg	HT RBS_ddc
53	ddc_fwd_5	gttgtgagggtaaacaactggcgtttaactttaagatgcttatatacatatg	HT RBS_ddc
54	ddc_fwd_6	gttgtgagggtaaacaactggcgtttaactttaagagactggtatacatatg	HT RBS_ddc
55	ddc_fwd_7	gttgtgagggtaaacaactggcgtttaactttaagacaggaatatacatatg	HT RBS_ddc
56	ddc_fwd_8	gttgtgagggtaaacaactggcgtttaactttaagagccccctatacatatg	HT RBS_ddc
57	ddc_fwd_9	gttgtgagggtaaacaactggcgtttaactttaagagcttattatacatatg	HT RBS_ddc
58	ddc_fwd_10	gttgtgagggtaaacaactggcgtttaactttaagaggattttatacatatg	HT RBS_ddc
59	ddc_fwd_11	gttgtgagggtaaacaactggcgtttaactttaagaatgagatatacatatg	HT RBS_ddc
60	ddc_fwd_12	gttgtgagggtaaacaactggcgtttaactttaagaaaacattatacatatg	HT RBS_ddc
61	ddc_fwd_13	gttgtgagggtaaacaactggcgtttaactttaagaaggagatatacatatg	HT RBS_ddc
62	ddc_fwd_14	gttgtgagggtaaacaactggcgtttaactttaagacccgaatatacatatg	HT RBS_ddc
63	ddc_fwd_15	gttgtgagggtaaacaactggcgtttaactttaagagcttggtatacatatg	HT RBS_ddc
64	ddc_fwd_16	gttgtgagggtaaacaactggcgtttaactttaagatggtattatacatatg	HT RBS_ddc
65	ddc_fwd_17	gttgtgagggtaaacaactggcgtttaactttaagacaggagtatacatatg	HT RBS_ddc
66	ddc_fwd_18	gttgtgagggtaaacaactggcgtttaactttaagaggcgactatacatatg	HT RBS_ddc
67	ddc_fwd_19	gttgtgagggtaaacaactggcgtttaactttaagaggggggtatacatatg	HT RBS_ddc
68	ddc_fwd_20	gttgtgagggtaaacaactggcgtttaactttaagaggcgtttatacatatg	HT RBS_ddc
69	ddc_fwd_21	gttgtgagggtaaacaactggcgtttaactttaagagggggttatacatatg	HT RBS_ddc
70	ddc_fwd_22	gttgtgagggtaaacaactggcgtttaactttaagaggctattatacatatg	HT RBS_ddc
71	ddc_fwd_23	gttgtgagggtaaacaactggcgtttaactttaagagggggctatacatatg	HT RBS_ddc
72	ddc_fwd_24	gttgtgagggtaaacaactggcgtttaactttaagaaggagatatacatatg	HT RBS_ddc
73	ddc_fwd_25	gttgtgagggtaaacaactggcgtttaactttaagaggggcgtatacatatg	HT RBS_ddc
74	ddc_fwd_26	gttgtgagggtaaacaactggcgtttaactttaagaacttcctatacatatg	HT RBS_ddc
75	ddc_rev	ccccgggtaccgagctcgacactagcccttgatcacgtcctg	HT RBS_ddc
76	Seq RBS hpa	ctgcttgccgaatatcatggtg	Sequencing RBS_hpaBC
77	Seq RBS ddc	tagtcagtggcgcgttcac	Sequencing RBS_ddc
78	Col_PCR_fwd	gctcgtataatgtgtggaattgtgagc	Quality check PCR
79	Col_PCR_rev	gcaaattctgttttatcagaccgc	Quality check PCR
80	tyrR-del-L5'	cgctgctcgtttgtctacccgcgcag	Host strain construction
81	tyrR-del-L3'-BamH	ttttggatcctgatatgacactatttggatagcaggaagg	Host strain construction
82	tyrR-del-R5'-BglII	ttttagatcttgcaacaccatcaggcatattaaattatgc	Host strain construction

83	tyrR-del-R3'	cattttcccgagtattgataccggtg	Host strain construction
84	Target uni-REV	actagtattatacctaggactgagctagc	Host strain construction
85	sgTyrR	tgtttggtcatgctccggaagttttagagctagaaatagcaagttaaaataaggctag	Host strain construction
86	sgXylAB	gcccaattcgctattccagcgttttagagctagaaatagcaagttaaaataaggctag	Host strain construction
87	XylA-int	gacgaactggtgttgggtaagcgtatggaagagcacttgcgttttgccgcctgct caaggcgcactcccgttctgg	Host strain construction
88	XylB-int	attaaagctgggacattgctcaggccggttaatttcgcggcccaatccagacacca gggttattgtctcatgagcg	Host strain construction
89	Xyl-scrin5’	taagtaacaatcaccgcgataaacgtaacc	Host strain construction
90	Xyl-scrin3’	tttatgttgctcatgccgagcgaaacaaac	Host strain construction

Supplementary Information S2:

The DNA sequences of the extracted genes were verified by sequencing and listed in this section.

DNA sequence of *hpaBC*:

ATGAAACCAGAAGATTTCCGCGCCAGTACCCAACGTCCTTTCACCGGGGAAGAGTATCTGAAAAGCCTGCAGGATGGTCG
CGAGATCTATATCTATGGCGAGCGAGTGAAAGACGTCACCACTCATCCGGCATTTCGTAATGCGGCAGCGTCTGTTGCCCA
GCTGTACGACGCACTGCACAAACCGGAGATGCAGGACTCTCTGTGTTGGAACACCGACACCGGCAGCGGCGGCTATACCC
ATAAATTCTTCCGCGTGGCGAAAAGTGCCGACGACCTGCGCCAGCAACGCGACGCCATCGCTGAGTGGTCACGCCTGAGC
TATGGCTGGATGGGCCGTACCCCAGACTACAAAGCCGCTTTCGGTTGCGCACTGGGCGCGAATCCGGGCTTTTACGGTCA
GTTCGAGCAGAACGCCCGTAACTGGTACACCCGTATTCAGGAAACTGGCCTCTACTTTAACCACGCGATTGTTAACCCACC
GATCGATCGTCATTTGCCGACCGATAAAGTGAAAGACGTTTACATCAAGCTGGAAAAAGAGACTGACGCCGGGATTATCG
TCAGCGGTGCGAAAGTGGTTGCCACCAACTCGGCGCTGACTCACTACAACATGATTGGCTTCGGCTCGGCACAAGTGATG
GGCGAAAACCCGGACTTCGCACTGATGTTCGTTGCGCCAATGGATGCCGATGGCGTGAAATTAATCTCCCGCGCCTCTTAT
GAGATGGTCGCGGGTGCTACCGGCTCGCCATACGACTACCCGCTCTCCAGCCGCTTCGATGAGAACGATGCGATTCTGGT
GATGGATAACGTGCTGATTCCATGGGAAAACGTGCTGATCTACCGCGATTTTGATCGCTGCCGTCGCTGGACGATGGAAG
GCGGTTTTGCCCGTATGTATCCGCTGCAAGCCTGTGTGCGCCTGGCAGTGAAATTAGACTTCATTACGGCACTGCTGAAAA
AATCACTCGAATGTACCGGCACCCTGGAGTTCCGTGGTGTGCAGGCCGATCTCGGTGAAGTGGTAGCGTGGCGCAACACC
TTCTGGGCATTGAGTGACTCGATGTGTTCAGAAGCAACGCCGTGGGTCAACGGGGCTTATTTACCGGATCATGCCGCACT
GCAAACCTATCGCGTACTGGCACCAATGGCCTACGCGAAGATCAAAAACATTATCGAACGCAACGTTACCAGTGGCCTGAT
CTATCTCCCTTCCAGTGCCCGTGACCTGAATAATCCGCAGATCGACCAGTATCTGGCGAAGTATGTGCGCGGTTCGAACGG
TATGGATCACGTCCAGCGCATCAAGATCCTCAAACTGATGTGGGATGCTATTGGCAGCGAATTTGGTGGTCGTCACGAACT
GTATGAAATCAACTACTCCGGTAGCCAGGATGAGATTCGCCTGCAGTGTCTGCGCCAGGCACAAAACTCCGGCAATATGG
ACAAGATGATGGCGATGGTTGATCGCTGCCTGTCGGAATACGACCAGGACGGCTGGACTGTGCCGCACCTGCACAACAAC
GACGATATCAACATGCTGGATAAGCTGCTGAAATAACGCAGCAGGAGGTTAAGATGCAATTAGATGAACAACGCCTGCGC
TTTCGTGACGCGATGGCCAGCCTGTCGGCAGCGGTAAATATTATCACCACCGAGGGCGACGCCGGACAATGCGGGATTAC
GGCAACGGCCGTCTGCTCGGTCACGGATACACCACCGTCGCTGATGGTGTGCATTAACGCCAACAGTGCGATGAACCCGG
TTTTTCAGGGCAACGGCAAGTTGTGCGTCAACGTCCTCAACCATGAGCAGGAACTGATGGCACGCCACTTCGCGGGCATG
ACAGGCATGGCGATGGAAGAGCGTTTTAGCCTCTCATGCTGGCAAAAAGGTCCGCTGGCGCAGCCGGTGCTAAAAGGTTC
GCTGGCCAGTCTTGAAGGTGAGATCCGCGATGTGCAGGCAATTGGCACACATCTGGTGTATCTGGTGGAGATTAAAAACA
TCATCCTCAGTGCAGAAGGTCATGGACTTATCTACTTTAAACGCCGTTTCCATCCGGTGATGCTGGAAATGGAAGCTGCGA
TTTGA

DNA sequence of *ddc*:

ATGCCCCCGAACAATTCCGCCAGTACGGCCACCAACTGATCGACCTGATTGCCGACTACCGCCAGACCGTGGGCGAACGC
CCGGTCATGGCCCAGGTCGAACCTGGCTATCTCAAGGCCGCCTTGCCCGCAACTGCCCCTCAACAAGGCGAACCTTTCGCG
GCCATTCTCGACGACGTCAATAACCTGGTCATGCCCGGCCTGTCCCATTGGCAGCACCCGGACTTCTATGGCTATTTCCCTT
CCAATGGCACCCTGTCCTCGGTGCTGGGGGACTTCCTCAGTACCGGTCTGGGCGTGCTGGGCCTGTCCTGGCAATCCAGCC
CGGCCCTGAGCGAACTGGAAGAAACCACCCTCGACTGGCTGCGCCAGTTGCTTGGCCTGTCTGGCCAGTGGAGTGGGGT
GATCCAGGACACTGCCTCGACCAGCACCCTGGTGGCGCTGATCAGTGCCCGTGAACGCGCCACTGACTACGCCCTGGTAC
GTGGTGGCCTGCAGGCCGAGCCCAAGCCTTTGATCGTGTATGTCAGCGCCCACGCCCACAGCTCGGTGGACAAGGCTGCA
CTGCTGGCAGGTTTTGGCCGCGACAATATCCGCCTGATTCCCACCGACGAACGCTACGCCCTGCGCCCAGAGGCACTGCA
GGCGGCGATCGAACAGGACCTGGCTGCCGGCAACCAGCCGTGCGCCGTGGTTGCCACCACCGGCACCACGACGACCACT
GCCCTCGACCCGCTGCGCCCGGTCGGTGAAATCGCCCAGGCCAATGGGCTGTGGTTGCACGTTGACTCGGCCATGGCCGG
TTCGGCGATGATCCTGCCCGAGTGCCGCTGGATGTGGGACGGCATCGAGCTGGCCGATTCGGTGGTGGTCAACGCGCAC
AAATGGCTGGGTGTGGCCTTCGATTGCTCGATCTACTACGTGCGCGATCCGCAACACCTGATCCGGGTGATGAGCACCAAT
CCCAGCTACCTGCAGTCGGCGGTGGATGGCGAGGTGAAGAACCTGCGCGACTGGGGGATACCGCTGGGCCGTCGGTTCC
GTGCGTTGAAGCTGTGGTTCATGTTGCGCAGCGAGGGTGTCGACGCATTGCAGGCGCGGCTGCGGCGTGACCTGGACAA
TGCCCAGTGGCTGGCGGGGCAGGTCGAGGCGGCGGCGGAGTGGGAAGTGTTGGCGCCAGTACAGCTGCAAACCTTGTG
CATTCGCCATCGACCGGCGGGGCTTGAAGGGGAGGCGCTGGATGCGCATACCAAGGGCTGGGCCGAGCGGCTGAATGC
ATCCGGCGCTGCTTATGTGACGCCGGCTACACTGGACGGGCGGTGGATGGTGCGGGTTTCGATTGGTGCGCTGCCGACC
GAGCGGGGGGATGTGCAGCGGCTGTGGGCACGTCTGCAGGACGTGATCAAGGGCTGA

Supplementary Information S3:

This overview provides the name of the constructs, along with the RBS sequences of *hpaBC* and *ddc*, and related dopamine concentration data.

Table 2: Library of different high throughput constructs and the different RBS sequences of *hpaBC* and *ddc*. Reference sequence is marked in red.

**Name**	**RBS_hpaBC**	**RBS_ddc**	**Dopamine [mg/L]**
**Ref**	AGGAGA	AGGAGA	59.86 ± 0.74
**A.01**	ACGTAC	TAGTCG	34.43 ± 2.72
**A.02**	GCTCAT	AAATAC	28.30 ± 0.99
**A.03**	CTCGGA	GTTGAG	54.73 ± 0.71
**A.04**	CGGGAG	ACTTCC	60.94 ± 3.64
**A.05**	CATCAG	TTCTTA	36.98 ± 2.34
**A.06**	AGGGGT	GACTGG	52.89 ± 0.77
**A.07**	CGCTAT	CAGGAA	40.37 ± 1.75
**A.08**	TAGGAC	GCCCCC	62.02 ± 1.47
**A.09**	TGGCAT	GCTTAT	48.00 ± 1.99
**A.10**	GGCAGG	GGATTT	69.03 ± 1.18
**A.11**	TAGGAG	ATGAGA	60.45 ± 2.17
**A.12**	AAGAGG	AAACAT	61.63 ± 2.66
**A.13**	GGCGAC	AGGAGA	60.64 ± 0.85
**B.01**	GGGGGC	CAGGAG	56.36 ± 1.24
**B.02**	GGGGGC	GGCGAC	26.44 ± 0.33
**B.03**	GGGGGC	GGGGGG	8.11 ± 0.55
**B.04**	GGGGGC	GGCGTT	53.57 ± 1.21
**B.05**	GGGGGC	AGGAGA	56.61 ± 1.81
**B.06**	GGGGGC	GGGGGT	6.98 ± 0.28
**B.07**	GGGGGC	GGCTAT	51.66 ± 2.02
**B.08**	GGGGGC	GGGGGC	10.47 ± 0.83
**B.09**	GGCGAG	GGGGGC	14.45 ± 0.10
**B.10**	GGCGAC	GGGGGC	12.03 ± 0.68
**B.11**	GGGGGG	GGGGGC	9.99 ± 0.16
**B.12**	GGCGTT	GGGGGC	12.55 ± 1.04
**B.13**	AGGAGA	GGGGGC	6.35 ± 1.24
**B.14**	GGGGGT	GGGGGC	11.17 ± 0.71

**B.15**	GGCTAT	GGGGGC	11.96 ± 0.28
**B.16**	AGGAGA	GGGGCG	3.68 ± 0.30
**B.17**	AGGAGA	ACTTCC	25.13 ± 3.49
**B.18**	GGGGCG	AGGAGA	52.94 ± 1.80
**B.19**	ACTTCC	AGGAGA	35.96 ± 0.22
**B.20**	GGGGCG	GGGGCG	28.10 ± 2.60
**B.21**	ACTTCC	ACTTCC	36.84 ± 1.05
**B.22**	GGGGCG	ACTTCC	47.02 ± 0.57
**B.23**	ACTTCC	GGGGCG	11.69 ± 2.80
**B.24**	GCTTGG	GCTTGG	45.31 ± 1.45
**B.25**	TGCTTA	TGCTTA	35.12 ± 0.67
**B.26**	TGGTAT	TGGTAT	43.71 ± 2.11
**B.27**	CCCGAA	GCTTGG	38.38 ± 0.65
**B.28**	GCTTGG	TGCTTA	41.95 ± 1.26
**B.29**	TGCTTA	TGGTAT	38.59 ± 1.62
**B.30**	TGGTAT	CCCGAA	41.95 ± 0.80
**B.31**	CCCGAA	TGCTTA	40.48 ± 1.21
**B.32**	GCTTGG	TGGTAT	10.12 ± 0.75
**B.33**	TGCTTA	CCCGAA	45.74 ± 1.38
**B.34**	TGGTAT	TGCTTA	38.46 ± 0.22
**B.35**	CCCGAA	TGGTAT	41.20 ± 3.58
**B.36**	GCTTGG	CCCGAA	40.54 ± 1.91
**B.37**	TGCTTA	GCTTGG	37.34 ± 0.94

Supplementary Information S4:

Figure 1 depicts that the majority of tyrosine is produced during the initial growth phase. Hence, *E. coli* FUS4.T2 was qualified as optimal host for the production of tyrosine-derived products.

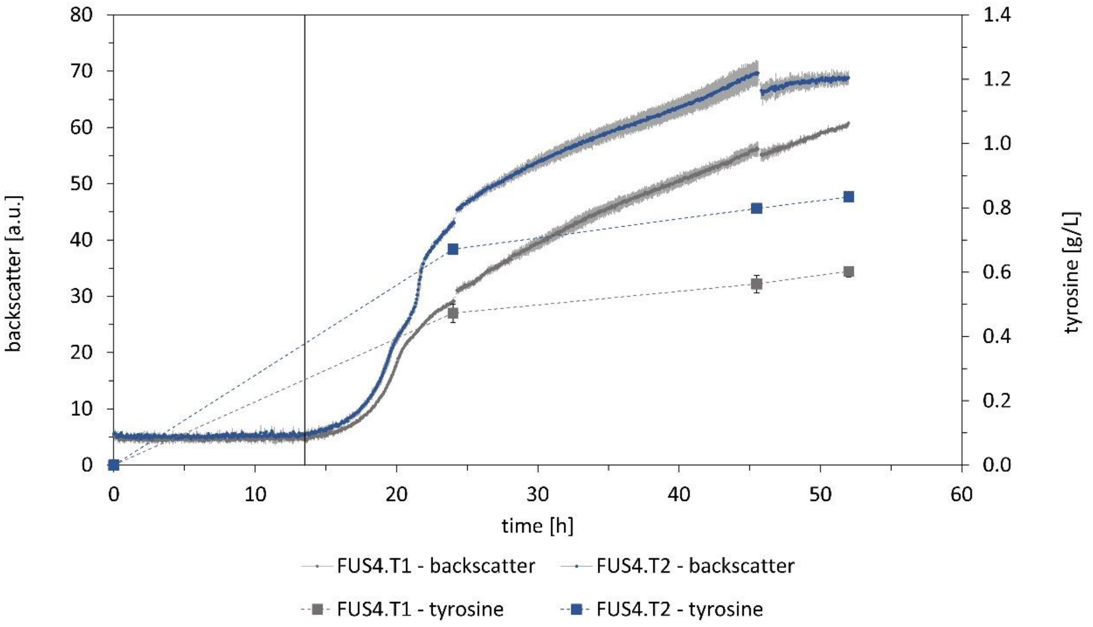

Figure 1: Host strain construction of *E. coli* FUS4.T1 and FUS4.T2. Microbioreactor cultivation of genome-edited *E. coli* strains for high tyrosine production. Automated inoculation after preculture reached backscatter 12 at approximately 13.5 h (indicated by black line). Cultivation was conducted in minimal medium. The strain *E. coli* FUS4.T1 is shown in grey whereas *E. coli* FUS4.T2 is shown in blue. Tyrosine was measured at specific time points (squares) whereas backscatter was measured continuously (lines). Data represent mean of replicates (*n*=3) with standard deviation as error bars.

Supplementary Information S5:

This section outlines our semi-automated workflow for the DBTL cycle. It includes details of the various operation units and the robot platforms used (Supplementary Figure 2). This workflow is designed to be flexible and can be combined as required.

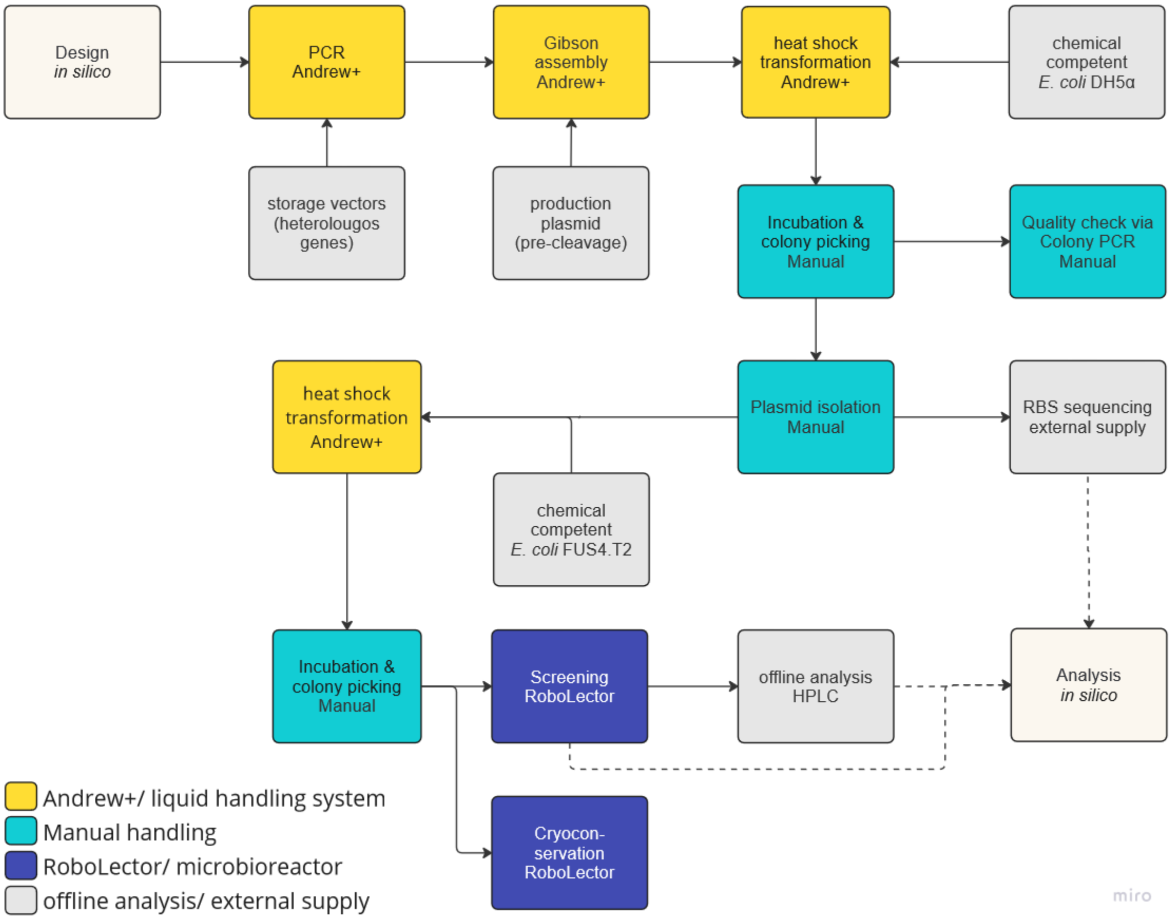

Figure 2: Overview of the semi-automated DBTL workflow. Each box describes the unit operation and the device for execution. Arrows indicate transfer of parts (solid) or data (dashed).

Supplementary Information S6:

Further details have been provided to facilitate a deeper comprehension of the underlying mechanisms involved in the design of heterologous dopamine pathways. Additionally, the impact of varying the GC content of RBS_hpaBC, while maintaining a constant GC content of RBS_ddc between 80 – 100 %, has been evaluated (Supplementary Figure 3).

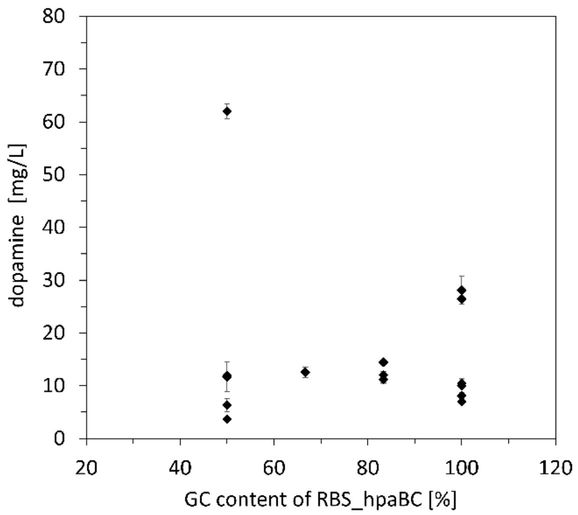

Figure 3: Evaluation of different RBS combinations. The GC content of RBS_ddc was kept at 80 – 100 % and different GC content of RBS_hpaBC was analysed. Data represent mean of replicates (*n*=3) with standard deviation as error bars.

Furthermore, the duplicates of the *in vitro* to *in vivo* mimicking experiment are presented to demonstrate the discrepancies within the same ratios (Supplementary Table 3). High differences between the single genes lead to high differences in dopamine production.

Table 3: Evaluation of the duplicates within every ddc:hpaBC ratio.

**TIR ratio (ddc x hpaBC)**	**TIR single hpaBC [%]**	**TIR single ddc [%]**	**dopamine [%]**
0.02	51.8	-51.8	53.6
0.05	9.8	-9.7	-32.4
0.1	13.3	-13.3	14.7
0.2	25.2	-25.2	33.2
0.333	14.9	-14.9	30.5
0.5	7.4	-7.4	-1.6

Supplementary Information S7:

Further details of the proteomic measurements were provided. While RBS engineering affects gene expression, selected samples were analysed for LC-MS/MS proteomics by external supply* (Supplementary Figure 4). We were able to show that increasing the GC content of RBS_ddc did not result in higher protein formation. On the contrary, increasing the strength of RBS_ddc led to a decrease in protein formation and therefore a decrease in dopamine production. For HpaBC, strong RBS_hpaBC led to high protein synthesis (Supplementary Table 4).

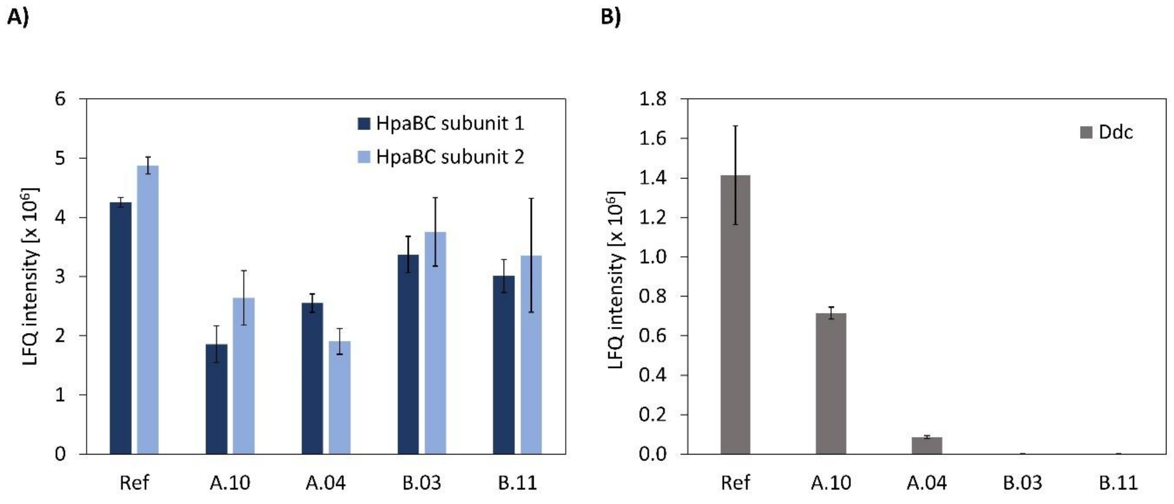

Figure 4: Proteomics data showing the expression level of A) enzyme HpaBC and B) Ddc in different engineered *E. coli* FUS4.T2 pJNTN_hpa_ddc. LFQ: label free quantification

However, both enzymes need to be balanced to maximise dopamine production. As A.10 resulted in the highest dopamine production even when both enzymes were not synthesised at maximum.

Table 4: Comprehensive data of the LC-MS/MS proteomics analysis*, compared with the GC content of the different RBS and the dopamine and L-DOPA concentration of each strain (*E. coli* FUS4.T2 pJNTN_hpa_ddc).

	Dopamine [mg/L]	L-DOPA [mg/L]	RBS_hpa GC_content [%]	rel_Prot_HpaBC [LFQ intensity x10^6]	RBS_ddc GC_content [%]	rel_Prot_Ddc [LFQ intensity x10^6]
**Ref**	59.86	0.00	50.0	4.26	50.0	1.414
**A.10**	69.03	0.00	83.3	1.86	33.3	0.715
**A.04**	60.94	6.10	83.3	2.55	50.0	0.086
**B.03**	8.11	108.24	100.0	3.37	100.0	0.001
**B.11**	9.99	112.55	100.0	3.01	100.0	0.001

(*LC-MS/MS data were measured by the Core Facility Hohenheim with the help of Jens Pfannstiel and Philipp Hubel.)
